# Detection of Certain Berries in Difficult Samples by Singleplex and Multiplex Real-Time PCR-HRM: A Case Study of Pitfalls

**DOI:** 10.3390/mps9020053

**Published:** 2026-04-01

**Authors:** Lenka Fialova, Ivana Marova

**Affiliations:** Faculty of Chemistry, Brno University of Technology, 612 00 Brno, Czech Republic; marova@fch.vut.cz

**Keywords:** plant DNA isolation, real-time PCR-HRM, multiplex PCR, berries, PCR inhibitors

## Abstract

Singleplex and multiplex real-time PCR-HRM (polymerase chain reaction with high resolution melting), both with specific and non-specific amplicon detection, are used for a wide range of applications, from clinical diagnostics to food authentication. However, their results can be influenced by the quality of the template DNA and composition of the reaction mixture. The methods used for the analysis of these results then influence the conclusions drawn. In this work we present an example from our laboratory practice, where the results of singleplex and multiplex real-time PCR differed, despite using the same reaction conditions, primers and analyzed plant material. We show the influence of a singleplex and multiplex PCR setup on the results, as well as the influence of template contamination on the melting behaviour of amplicons. We also discuss the usefulness of cluster analysis for the clarification of real-time PCR-HRM results which appear unclear when only melting and difference curves or similarity scores are used for the analysis of these results. We provide a discussion of problems which we encountered during an analysis of commercial teas and which should be considered by researchers new to PCR-based analysis of plant material, especially if the studied material is rich in various contaminants.

## 1. Introduction

In recent years, several multiplex real-time PCR-HRM assays, mainly for the detection and identification of several bacterial species in clinical samples, have been developed [1,2,3,4]. These assays combine two analytical methods, multiplex PCR and real-time PCR-HRM, thereby saving time and reagents. Both methods are already well established and numerous articles presenting their various applications have been published. Multiplex PCR has been used, e.g., for clinical diagnostics [5], the detection of pathogenic bacteria in seafood [6] and the authentication of food [7,8]. HRM analysis has been used, e.g., for the differentiation of *Shigella* species found in food and stool [9], various clinical studies [10,11,12], food authentication [13,14] and many other applications. However, the multiplex real-time PCR-HRM approach is less common. In articles focusing on multiplex PCR assays, amplicon detection is often carried out either by horizontal agarose gel electrophoresis [6,15,16,17] or, in the real-time version, by specific fluorescent probes [18,19,20,21]. High-resolution melting assays are usually done following a singleplex PCR reaction. However, multiplex real-time PCR-HRM assays with non-specific amplicon detection have recently been developed, again for analysis of clinical samples and animal-based foods [22,23,24].

Multiplex PCR is based on the same principle as singleplex PCR. The main difference between a singleplex and a multiplex PCR assay is the presence of two or more primer pairs in the PCR mixture, compared to singleplex PCR, where only one primer pair is present [25]. The main advantage of multiplex PCR is the saving of time, materials and energy [25]; for example, in a multiplex PCR assay, it is possible to detect multiple targets in the time that one target would be detected if singleplex PCR was used. The main disadvantage of multiplex PCR is that method optimization is difficult, as the same guidelines for primer design which apply to singleplex PCR apply for multiplex PCR, too. Additionally, attention should be paid to the difference in the size of amplicons and cross-amplification [25].

HRM analysis is a post-PCR method which distinguishes between amplicons based on their melting behaviour and can detect even single-nucleotide differences [26]. To carry out HRM analysis, the target sequence must be amplified in the presence of a fluorescent saturation dye. Saturation dyes can be used in high concentrations without inhibiting amplification, thus making it possible to label the amplicons along their entire length [27]. The outputs of HRM analysis are melting curves, which are obtained by plotting fluorescence intensity against temperature. These raw data need to be normalized to remove both the effect of background fluorescence and the differences caused by other factors, such as pipetting [26,27].

To get an accurate result from HRM analysis, the melting curves must be normalized correctly. The regions of the melting curves which are considered 100% and 0% of fluorescence intensity should not encroach on the active melt phase of the melting curve. However, correct data normalization does not absolutely guarantee that the result of the analysis will be accurate. Slomka et al., in their 2017 article [28], summarize factors which can influence the result of HRM analysis even to the point of it being incorrect. These factors include different DNA isolation protocols [29], the fluorescent dyes used for the analysis [30], primer design [28], etc.

This work is an offshoot of experiments which we performed during the development of a PCR-based method for analysis of plant-based foods. We, like Slomka et al. in their 2017 paper [28], aim our work mainly at researchers new to real-time PCR-HRM-based analyses. We present issues and pitfalls which we have encountered in our laboratory practice, starting with template quality, continuing with the PCR reaction setup and ending with data analysis. We address the melting behaviour of amplicons in singleplex and multiplex PCR and the influence of a contaminated template on the result of HRM analysis, as well as the possible ways to analyse the data and their suitability for the analysis of real-time PCR with a contaminated template. The samples analyzed in this work are commercial teas (fruit and herbal), which are an example of difficult samples, both because of the way they are usually stored (long-time storage at room temperature) and because of their composition. The teas used in this work contain plant species such as hibiscus, apple and rose (in the form of rosehips), all of which are known to contain high levels of polysaccharides and phenolic compounds [31,32,33]. These substances are known inhibitors of PCR, and they are also notoriously difficult to remove during DNA purification. In the conclusion of this work, we also offer a set of practical suggestions for the design of real-time PCR-based assays meant for analysis of contaminated samples, as, to our knowledge, such suggestions are extremely useful but at the same time are often difficult to find in the literature.

## 2. Materials and Methods

### 2.1. Samples for DNA Extraction

Raspberry, blackberry, and blueberry leaves were used as reference samples in this work. Regarding commercial samples, five commercial bagged teas were analyzed in this work. All teas and dried blackberry leaves were obtained from local supermarkets and drugstores in Brno, Czech Republic, and stored at room temperature. Raspberry and blueberry leaves were obtained from private gardens of the authors’ coworkers. These leaves were lyophilized and stored at −20 °C. Each of these teas contained at least one out of the three above-mentioned fruit species (raspberry, blackberry, and blueberry). The detailed composition of the commercial teas (as declared by the manufacturers) is shown in Table 1.

### 2.2. DNA Extraction from Reference Samples

Briefly, DNA was extracted from blueberry, raspberry and blackberry leaves using the EliGene^®^ Plant DNA Isolation Kit (Elisabeth Pharmacon, Brno, Czech Republic). The isolation was done according to the manufacturer’s instructions. The concentration and purity of isolated DNA was determined by UV–VIS spectrophotometry with the NanoDrop 2000 (Thermo Scientific, Waltham, MA, USA). Each DNA preparation was measured four times.

### 2.3. DNA Extraction from Commercial Teas

DNA was extracted from dry commercial fruit tea mixtures using a modified version of the protocol described by Glyn et al. [34]. Briefly, 0.05 g of each dry sample was incubated for 30 min with 1 mL of CTAB (cetyltrimethylammoniumbromide) buffer [35], to which 1 µL of β-mercaptoethanol was added. After the incubation, crude contaminants were removed by centrifugation at 10,000× *g* for 10 min. DNA was purified with chloroform:isoamylalcohol (24:1), followed by centrifugation at 10,000× *g* for 10 min. The water phase was transferred into a new Eppendorf tube, and it was mixed with 200 μL of 300 mM calcium chloride and incubated at room temperature for 15 min. This step was followed by isopropanol precipitation. The precipitated DNA was incubated with 0.5 mL of 10 nM ammonium acetate in 70% ethanol, followed by centrifugation (5 min at 10,000× *g*). The supernatant was removed, and the sediment was resuspended in 250 μL of 1× TE (Tris-EDTA) buffer. A total of 250 µL of 5 M NaCl and 1250 µL of cold 99% ethanol was added. The sample was mixed by inversion and incubated for 15 min at −20 °C to facilitate the precipitation of DNA. The incubation was followed by centrifugation (15 min at 10,000× *g*). The DNA in the sediment was dissolved in 100 µL of 1× TE buffer and its concentration and purity were determined by ultraviolet (UV–VIS) spectrophotometry using the NanoDrop 2000 (Thermo Scientific).

### 2.4. Primers

All primer sequences used in this work were retrieved from the literature [36,37]. The primers were synthetized by Generi Biotech (Hradec Kralove, Czech Republic). The primer sequences used in this work are summarized in Table S1.

### 2.5. Singleplex Real-Time PCR

All singleplex PCR reactions were carried out in LightCycler Nano (Roche, Basel, Switzerland). All PCR assays were performed using the same PCR profile (95 °C/5 min, followed by 40 cycles of 95 °C/20 s, 59 °C/15 s and 65 °C/60 s, and concluded by 72 °C/5 min). All PCR mixtures were prepared using the SYTO9 MasterMix (Top Bio, Vestec, Czech Republic) according to the manufacturer’s instructions. All primers were diluted to 10 pmol/μL solutions. The estimated amount of DNA for each reaction was 10 ng. The PCR reactions were carried out in a total volume of 25 µL. The detailed compositions of the PCR mixtures for singleplex PCR are in Table S2. All singleplex PCR analyses were performed in three technical replicates.

### 2.6. Multiplex Real-Time PCR

All multiplex PCR reactions were carried out using the same instrumentation and PCR profile as the singleplex PCR reactions. The same reaction components were used and the primer and SYTO9 MasterMix concentrations were the same as for the singleplex PCR reactions. The estimated amount of DNA for each reaction was also 10 ng. The reactions were also carried out in a total volume of 25 µL. The detailed composition of the reaction mixture for multiplex PCR is described in Tables S3 and S4. All multiplex PCR analyses were performed in four technical replicates due to concerns related to the possibly greater sensitivity to inhibition.

### 2.7. High-Resolution Melting Analysis (HRM)

High-resolution melting analysis was carried out in LightCycler Nano (Roche). The starting temperature was 60 °C and the end temperature was 97 °C with 0.05 °C intervals. The melting curves were analyzed using the software LightCycler Nano SW 1.1. The curves of VcBHLH003 amplicons (blueberry) were normalized in the region of 79.5–84.5 °C and the curves of RiACO1 amplicons (raspberry and blackberry) were normalized in the region of 83.5–87.0 °C.

### 2.8. Statistical Analysis

As an additional way of verifying the results, the normalized fluorescence values were used to build similarity matrices. Like Nunziata et al. [38], genotype confidence percentages were used as similarity values. These values were calculated according to the formula used by Nunziata et al. [38]:

(1) $$S_{rt}=\;1.05^{\left[-0.02\times \sum_{i=a}^{z}\left(f_{ri}-\;f_{ti}\right)2\right]}$$

where *a* is the temperature at the start of melting and *z* is the temperature at the end of melting, *f_ti_* and *f_ri_* are the normalized fluorescence values of compared samples *r* and *t* detected at temperature *i*, and the constants 1.05 and −0.02 are the spread factor and sharpness factor, respectively [39].

Cluster analysis of RiACO1 amplicons was also performed using the software Orange Data mining (version 3.38.1), with Euclidean distances used to build dissimilarity matrices and Ward linkage to plot the dendrograms. Normalized fluorescence values from the temperature range of 83.5–87.0 °C were used as input data. Data normalization was performed using the Roche LightCycler Nano software.

## 3. Results

### 3.1. DNA Concentration and Purity

The DNA concentration ranged from 45.7 ± 0.8 ng∙µL^−1^ (tea no. 4, Table 2) to 124.5 ± 0.5 ng∙µL^−1^ (tea no. 2). It is, however, possible that the DNA concentrations measured in teas no. 1, 2 and 3 were overestimated, as these samples also showed significant contamination by both proteins and phenolic compounds (Table 2).

**Figure 1:**
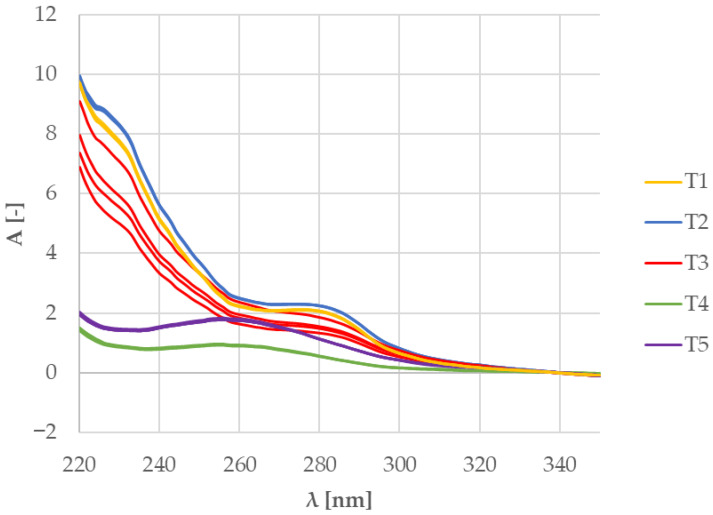
Absorption spectra of DNAs isolated from commercial samples. T1 = Tea 1, T2 = Tea 2, T3 = Tea 3, T4 = Tea 4, and T5 = Tea 5.

Regarding the purity of the isolated DNA, a relationship can be seen between the A260/A280 and A260/A230 absorbance ratios (Table 2), the shapes of UV absorption spectra (Figure 1) and the composition of the samples (Table 1).

The samples which showed the highest levels of contamination (teas no. 1, 2 and 3; Table 2) all contained a significant quantity of both rosehips and hibiscus blossoms (Table 1). All DNA isolates from teas 1, 2 and 3 showed high absorbance in the region between 220 and 260 nm. This, together with the A260/A230 ratio, suggests phenolic contamination. Because our isolation protocol does not use phenol, the contamination likely comes from the plant material. The differing levels of contamination between teas 1, 2, and 3, and teas 4 and 5 may be explained by the presence of rosehips in teas 1, 2 and 3, and their absence in teas 4 and 5, because teas 4 and 5, which did not contain rosehips, showed lower levels of phenolic contamination. We would also like to point out the behaviour of sample no. 3, which showed higher variation in concentration measurements compared to the remaining samples (Table 2 and Figure 1).

### 3.2. Multiplex vs. Singleplex: Influence of Reaction Mixture Composition on Results of HRM Analysis—Primers VcBHLH003 (Blueberry-Specific)

**Figure 2:**
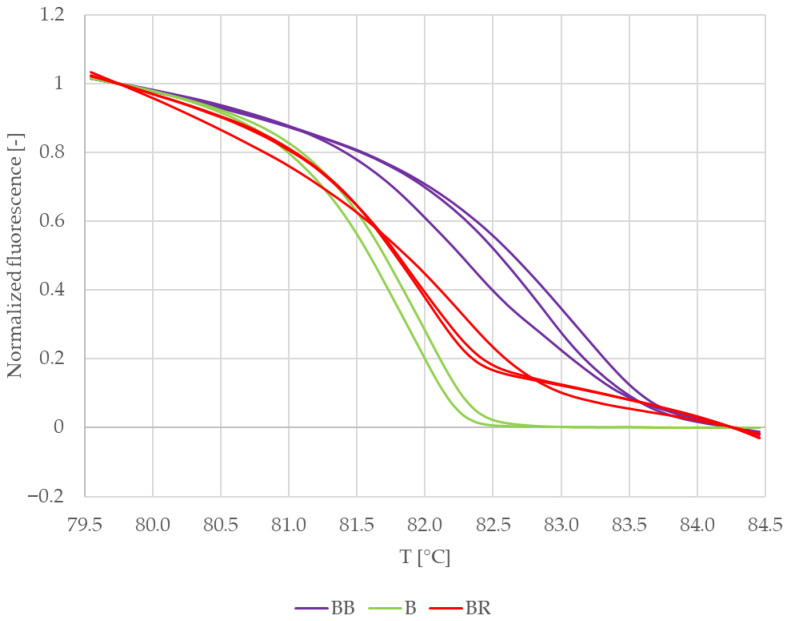
Normalized melting curves of reference samples—blueberry leaves (VcBHLH003 amplicons). BB = multiplex PCR mixture with blueberry and blackberry DNA, B = singleplex PCR mixture, and BR = multiplex PCR mixture with blueberry and raspberry DNA.

Figure 2 shows the normalized melting curves of VcBHLH003 amplicons clustered to three distinctive groups, even though DNA from the same plant and the same preparation was used as a template. It is therefore possible that the melting behaviour of VcBHLH003 amplicons was influenced by the reaction mixture composition.

**Figure 3:**
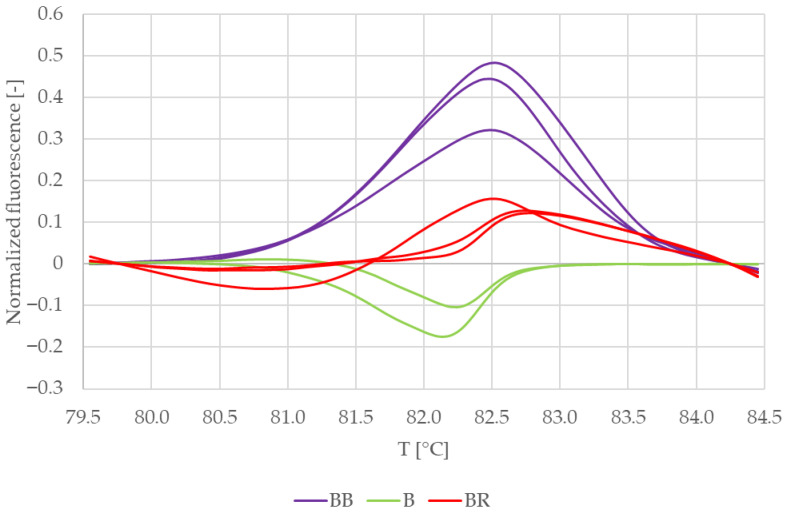
Difference plots of reference samples—blueberry leaves (VcBHLH003 amplicon). BB = multiplex PCR mixture with blueberry and blackberry DNA, B = singleplex PCR mixture with blueberry DNA, and BR = multiplex PCR mixture with blueberry and raspberry DNA.

Difference plots of the normalized melting curves were constructed to clarify the result (Figure 3). The curves in Figure 3 are clustered in two distinct groups. The curves representing VcBHLH003 amplicons from duplex PCR with blackberry DNA as a template for RiACO1 primers (purple) make up one group, while the second group consists of curves representing VcBHLH003 amplicons obtained by singleplex PCR (green) and duplex PCR with raspberry DNA as a template for RiACO1 primers (red). Based on Figure 3, a conclusion may be drawn that the composition of the reaction mixture had an influence only on the melting behaviour of VcBHLH003 amplicons obtained in duplex PCR with blackberry DNA as a template for RiACO1 primers.

Because the conclusions drawn based on Figure 2 and Figure 3 were contrary, the result was further verified by building a similarity matrix. Genotype confidence percentages were used as similarity values and were calculated according to the formula shown in Section 2.7. Table 3 summarizes the average similarity values between replicate samples and between VcBHLH003 amplicons obtained by singleplex and multiplex PCR.

The similarity values for replicate samples were higher than 98% for all sets of VCBHLH003 amplicons. Regarding the similarity values among the three sample sets, the amplicons obtained in singleplex PCR showed a similarity higher than 99% when compared to the amplicons obtained in duplex PCR with raspberry DNA as a template for RiACO1 primers. Amplicons obtained in duplex PCR with blackberry DNA as a template for RiACO1 primers showed 96.87% similarity to amplicons obtained in duplex PCR with raspberry DNA as a template for RiACO1 primers, while the similarity to the amplicons obtained by singleplex PCR was lower than 95%.

### 3.3. Multiplex vs. Singleplex: Influence of Reaction Mixture Composition on Results of HRM Analysis—Primers RiACO1

**Figure 4:**
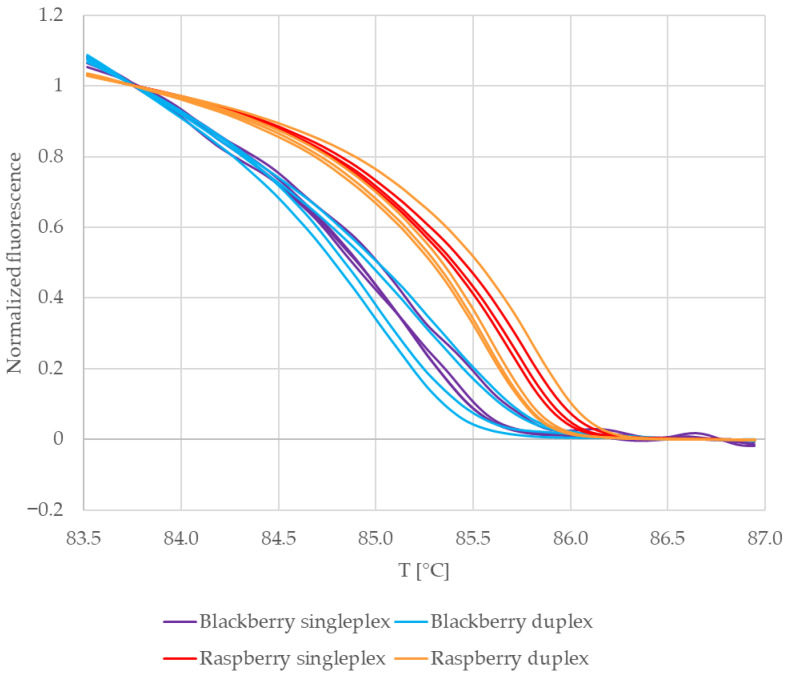
Normalized melting curves of reference samples (blackberry and red raspberry leaves).

In the case of RiACO1 amplicons, four sets of replicate samples were analyzed. The first two sets were amplicons from singleplex PCR. One set contained raspberry DNA, and the second set contained blackberry DNA. The next two sets were amplicons from multiplex PCR. One set contained blueberry and blackberry DNA, while the other set contained blueberry and raspberry DNA. The normalized melting curves of these amplicons are shown in Figure 4.

**Figure 5:**
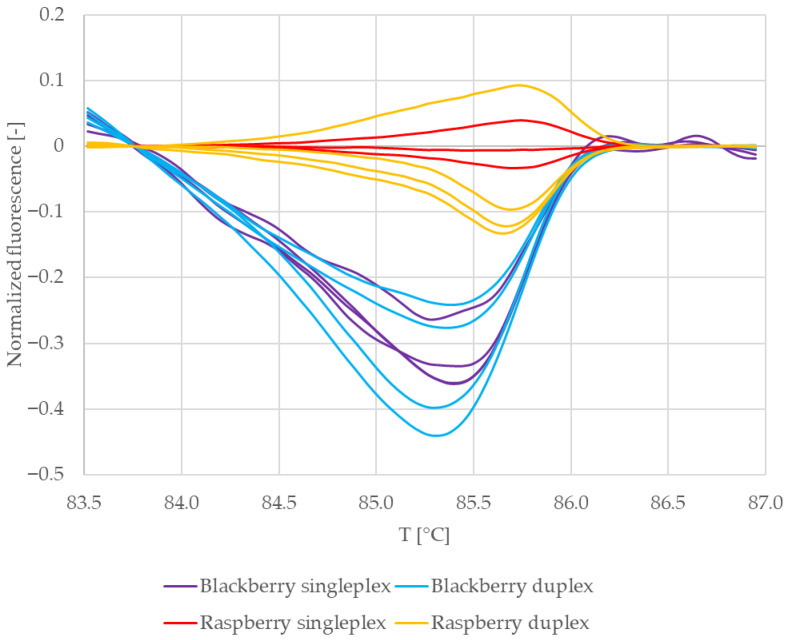
Difference plots of reference samples (blackberry and red raspberry leaves).

The melting curves representing amplicons which had raspberry DNA as a template are clustered together, and the same is true for amplicons which had blackberry DNA as a template. Furthermore, the curves representing raspberry DNA are clearly separated from the curves representing blackberry DNA. This is also visible in the difference plots of the normalized melting curves (Figure 5). All curves representing amplicons with raspberry DNA as a template (red and yellow) are clustered around the baseline, while all curves representing amplicons with blackberry DNA as a template (purple and blue) form a separate group. It may therefore be said that in the case of multiplex PCR, the composition of the reaction mixture had no significant influence on the melting behaviour of the RiACO1 amplicons.

This result was further verified by building a similarity matrix using the normalized fluorescence data. The average similarity values (expressed as percentages) are shown in Table 4. First, replicate samples in each set were compared with each other. In the case of both raspberry and blackberry DNA, the replicate samples showed a similarity higher than 99% in both singleplex and multiplex PCR (Table 4, lines no. 1–3 and 5). Next, amplicons obtained in singleplex PCR were compared to amplicons obtained in multiplex PCR. In the case of both raspberry and blackberry DNA, the similarity values were higher than 99% (Table 4, lines no. 4 and 6), which confirmed that the reaction mixture composition (singleplex vs. multiplex PCR) had no influence on the melting behaviour of the RiACO1 amplicons.

Finally, amplicons with raspberry DNA as a template were compared to amplicons with blackberry DNA as a template (Table 4, lines no. 7–10). The similarity values between these samples ranged from 95.47% to 96.43%. This meant that the threshold for confirmation of either one or the other species had to be set at a higher value. The threshold value of 99% was supported both by the similarity score between raspberry and blackberry calculated in this work and by the result of multiple sequence alignment performed in NCBI BLAST (version) 2.15.0, which shows 98.31% similarity between the RiACO1 amplicon of raspberry DNA and cloudberry DNA (Supplementary File S3). A raspberry 1-aminocyclopropane-1-carboxylate oxidase sequence (accession no. KP125887.1), more precisely the 177 bp region amplified by the RiACO1 primers (from position no. 421 to 597), was used as input data. A threshold of 99% was set to reflect the similarities between raspberry and the two other *Rubus* species. A similarity score value in the 95–99% range was considered inconclusive (i.e., *Rubus* DNA is present, but the particular species cannot be confirmed) on the basis of the similarity score calculated here and of the BLAST alignment result mentioned above.

### 3.4. Commercial Teas: Detection of Rubus Species by Singleplex Real-Time PCR-HRM

RiACO1 amplicons were detected in teas no. 1–4, all of which had raspberry and/or blackberry declared in their composition. RiACO1 amplicons were not detected in sample no. 5, which had neither raspberry nor blackberry declared in its composition (Table 1). The RiACO1 amplicons underwent HRM analysis as described in Section 2.7 and their melting curves were normalized.

**Figure 6:**
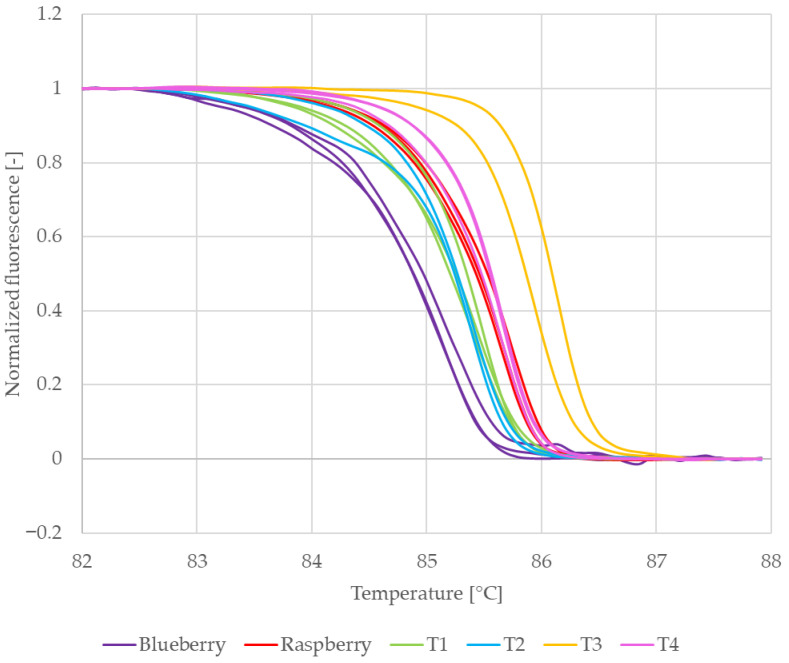
Normalized melting curves of singleplex PCR (primers RiACO1) results with commercial teas. T1 = Tea 1, T2 = Tea 2, T3 = Tea 3, and T4 = Tea 4.

Figure 6 shows the normalized melting curves of the commercial teas and reference samples in which RiACO1 amplicons were detected. Curves belonging to raspberry leaves (red) and blackberry leaves (dark purple) are clearly separated. Curves belonging to tea no. 4 (pink) are clustered together with curves belonging to raspberry leaves. This would suggest that in tea no. 4, raspberry DNA was amplified. Curves which represent teas no. 1 and 2 are clustered together and lay between curves which belong to both reference samples. That would indicate that in these teas, a mixture of raspberry and blackberry DNA was amplified. Melting curves representing tea no. 3 lay separately from other commercial and reference samples. This would indicate that tea no. 3 possibly contained DNA from a *Rubus* species other than raspberry or blackberry. However, the only *Rubus* species declared in the composition of this tea (Table 1) is raspberry. When taken together with the behaviour of the DNA sample from this tea during spectrophotometric measurements (low DNA purity combined with significantly lower reproducibility than the rest of the samples), a conclusion may also be drawn that raspberry DNA was amplified, but the behaviour of the amplicon during melting was influenced by contaminants present in the analyzed DNA.

**Figure 7:**
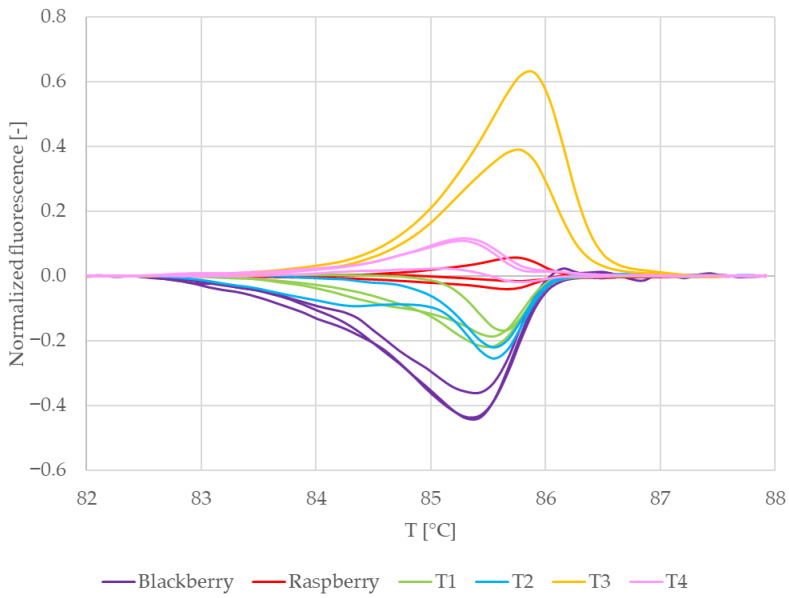
Difference plots of commercial samples (RiACO1 amplicons). T1 = Tea 1, T2 = Tea 2, T3 = Tea 3, and T4 = Tea 4.

**Figure 8:**
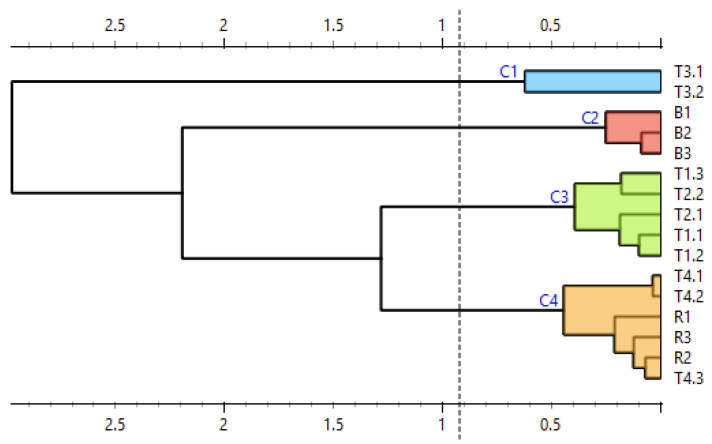
Similarity of commercial and reference samples, presented as a dendrogram. B = blackberry, R = raspberry, T1 = tea 1, T2 = tea 2, T3 = tea 3, and T4 = tea 4.

To further clarify the results, difference plots of normalized curves were constructed (Figure 7). Similarly to Figure 6, Figure 7 shows curves representing raspberry (red) and tea no. 4 (pink) clustered together and curves representing tea no. 3 (yellow) laying separately from both reference and other commercial samples. The curves representing teas no. 1 and 2 lay between the curves representing raspberry and blackberry. Their shape is similar to the shape of the curves representing blackberry, and in the region between ca. 85.5 °C and 86.5 °C, they partially overlap these curves. This means that the difference plots support the conclusion drawn based on the shapes of the normalized melting curves. For further and final confirmation, we built a similarity matrix with similarity scores calculated according to the formula in Section 2.7. We also performed cluster analysis using the Euclidean distance between samples as a distance metric and Ward linkage. The similarity values of commercial and reference samples are shown in Table 5. The results of the cluster analysis are shown in Figure 8.

Out of the three teas in which raspberries were declared (T1, T3 and T4), raspberry DNA was confirmed in one (tea T4): the similarity score between its RiACO1 amplicons and the RiACO1 amplicons of raspberry DNA was higher than 99% (Table 5). Tea T4 was also grouped together with raspberry in the cluster analysis (Figure 8). In the case of tea T3, the similarity between the commercial tea and raspberry reference sample was 97.44%, while the similarity with the blackberry sample was 90.92%, and the cluster analysis grouped this tea separately from both reference samples and other commercial teas (Figure 8). This result could be interpreted in two ways: The first interpretation is that tea T3 did not contain either blackberry or raspberry DNA, but DNA from other *Rubus* species was present. The second interpretation is that raspberry DNA may have been amplified (the similarity value between sample T3 and raspberry DNA is higher than the similarity between sample T3 and blackberry DNA), but the melting behaviour of the amplicon was somehow influenced. It is likely that this result is connected to the contamination in the template DNA, as certain types of contaminants can influence the melting behaviour of the amplicon. While teas T1 and T4 showed DNA contamination (Table 2), the variation between individual concentration measurements was 0.6 and 0.8 ng∙µL^−1^, respectively. The behaviour of tea T3 during DNA concentration measurements was, however, different. DNA from tea T3 showed higher variation between individual measurements (13.3 ng∙µL^−1^), as shown in Table 2 and Figure 1. Each of these three teas (T1, T3 and T4) had 2% raspberries declared in their composition, but raspberry DNA was confirmed only in tea T4. While the presence of raspberry DNA was not absolutely confirmed in tea T1 (this sample is not grouped together with raspberry and tea T4), the results indicate the presence of related DNA and the sample showed consistent behaviour between replicates. However, tea T3 showed more variance between replicates than teas T1 and T4 (Figure 7 and Figure 8).

In the case of tea T2, the result was also ambiguous. The similarity value between this sample and blackberry DNA was higher than 99%, but the same was true for the similarity value between tea T2 and raspberry DNA, even though only blackberries were declared in this sample.

### 3.5. Commercial Teas: Detection of Rubus Species by Multiplex Real-Time PCR-HRM

**Figure 9:**
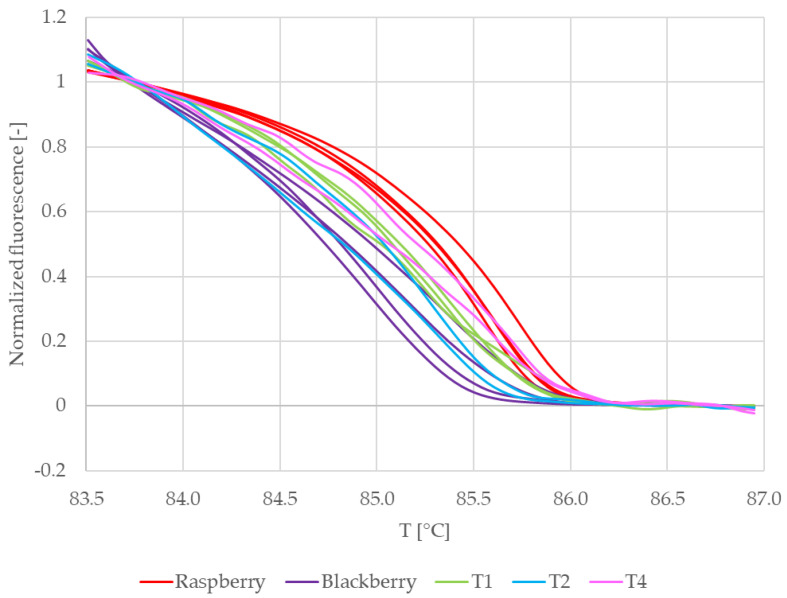
Normalized melting curves—duplex PCR (RiACO1 amplicons) with commercial samples. T1 = Tea 1, T2 = Tea 2, and T4 = Tea 4.

**Figure 10:**
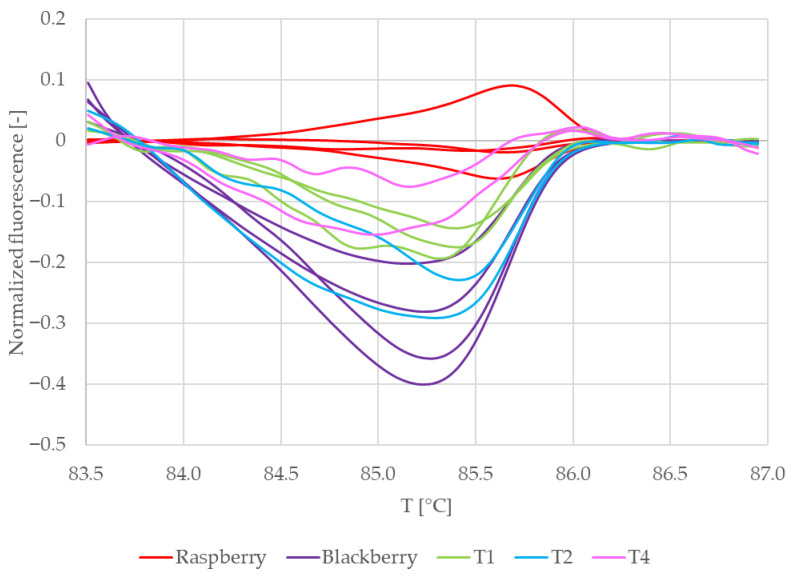
Difference plots—duplex PCR (RiACO1 amplicon) with commercial samples. T1 = Tea 1, T2 = Tea 2, and T4 = Tea 4.

**Figure 11:**
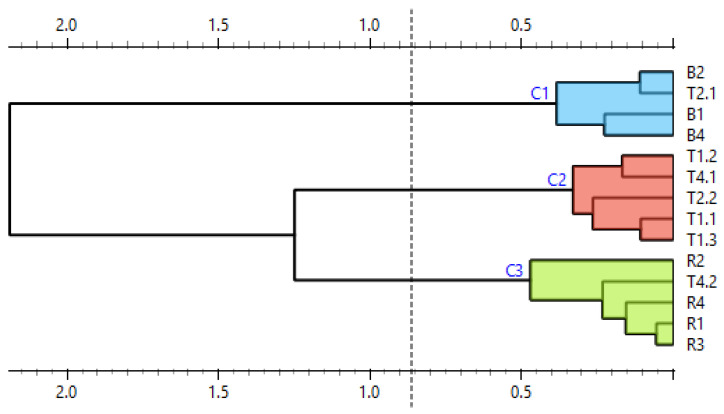
Similarity of reference and commercial samples represented as a dendrogram (result of multiplex PCR). B = blackberry, R = raspberry, T1 = tea 1, T2 = tea 2, T3 = tea 3, and T4 = tea 4.

RiACO1 amplicons were detected in teas no. 1, 2, and 4. The amplicons underwent HRM analysis with the same conditions as the RiACO1 amplicons obtained in singleplex PCR. Figure 9 shows the normalized melting curves, while their difference plots are shown in Figure 10. As was the case with the results of singleplex PCR, the curves representing raspberry (red) and blackberry (purple) are separated and the curves representing tea no. 1 (green) lay between them, indicating that this tea possibly contained both raspberry and blackberry DNA, which is a result consistent with the tea’s declared composition (Table 1). In case of teas no. 2 (blue) and 4 (pink), the result of multiplex PCR differs from the result of singleplex PCR. In the case of singleplex PCR, the curves representing tea no. 2 showed consistent behaviour of replicates: they overlap with curves representing tea no. 1 and are also clustered together in the dendrogram (Figure 8). The result of multiplex PCR for tea no. 2 shows inconsistent behaviour between replicates (an overlap with both blackberry and tea no. 1, while the singleplex PCR result shows an overlap with tea no. 1 only). The replicates of this sample (tea no. 1) can also be found in different clusters of the dendrogram, while after singleplex PCR, they are in the same cluster (Figure 8 and Figure 11). In the case of tea no. 4, the melting curves are clearly grouped together with the curves representing raspberry, which was also confirmed by the cluster analysis (Figure 7 and Figure 8). However, in the case of multiplex PCR, an overlap with both tea no. 1 and raspberry is visible and the replicates of tea no. 4 are placed in different clusters of the dendrogram. DNA from tea no. 3, where amplicons with inconsistent behaviour were detected in singleplex PCR, was not amplified using multiplex PCR.

For further clarification of the result, difference plots of the normalized melting curves were constructed (Figure 10). The shape of the curves representing tea no. 4 differs from the shape of the curves obtained by analysis of singleplex PCR amplicons (Figure 7). It also differs from the shapes of curves belonging to other reference samples and commercial teas, and the possible influence of background noise is visible (the curves are wavy instead of smooth). It is therefore difficult to conclude if tea no. 4 contained raspberry or blackberry DNA.

To further clarify the results, a similarity matrix was built and cluster analysis was performed. The similarity values between the commercial teas and the reference samples are shown in Table 6, and the results of the cluster analysis are shown in Figure 11. As mentioned earlier, using multiplex PCR, RiACO1 amplicons were detected in teas T1, T2 and T4. Raspberry and/or blackberry was declared by the manufacturer in all these teas. In the case of tea T1, the similarity value between this sample and raspberry DNA was 99.24% and the similarity value between tea T1 and blackberry DNA was 99.05% (Table 6)—which is a result similar to the result of the corresponding singleplex PCR assay. The cluster analysis placed this tea into a group separate from both raspberry and blackberry, with a closer relation to raspberry (Figure 11). This result agrees with the result of the singleplex PCR. In the case of tea T2, the similarity between the DNA from this tea and blackberry DNA was 99.68%, meaning that according to the similarity score, blackberry DNA was amplified. The similarity between tea T2 and raspberry DNA was 97.69%, which would indicate that raspberry DNA was not present in the sample. This result agrees with the description of tea T2: only blackberries are declared in its composition. However, this result differs from the one obtained by HRM analysis of amplicons obtained by singleplex PCR. When analyzed further using the cluster analysis, inconsistencies in the behaviour of replicate samples are also evident (Figure 11). In the case of tea T4, when only the similarity scores (Table 6) are considered, the result obtained by multiplex PCR matches the result obtained by singleplex PCR: raspberry DNA was amplified, while blackberry DNA was not. However, when the results of the cluster analysis (Figure 11) are also considered, tea T4, similarly to tea T2, also shows inconsistent behaviour.

To sum up, the results of the analysis of tea T1 obtained by multiplex and singleplex PCR matched. In the case of teas T2, T3 and T4, the results did not match. Tea T3, in which we observed a possibly altered amplicon melting behaviour in singleplex PCR and inhibition of amplification in multiplex PCR, was already problematic during DNA concentration measurements. As shown in Table 2 and Figure 1, the variability in concentration measurements of DNA from tea T3 was higher than the variability in the rest of the samples. The behaviour of DNA from this tea in both PCR analyses and during concentration measurements may be explained by the tea’s composition: the first four (and therefore the four most abundant) components of this sample are rosehip peels, hibiscus blossoms, apple fruit and orange peels. Two of these components (rosehip and hibiscus) are rich in phenolic compounds, and all four of these components are rich in polysaccharides such as pectin, which is a known PCR inhibitor. In comparison, teas T1, T2 and T4, which showed consistent behaviour both during concentration measurements and in singleplex PCR (in the case of tea T1, also in multiplex PCR), contained fewer problematic components among the four which were most abundant, such as blackberry leaves, chamomile blossom or seaberry fruit instead of rosehip, orange or apple. In the case of tea T4, the results obtained by singleplex PCR and multiplex PCR did not match: the result of singleplex PCR confirmed the presence of raspberry DNA and the behaviour of replicate samples was consistent, while the result of multiplex PCR showed inconsistent behaviour of replicate samples (Figure 11). The behaviour of DNA from tea T2 in multiplex PCR was similar to that of DNA from T4.

Several conclusions may be drawn based on the results shown. First, relatively high levels of organic contamination (with an A260/A230 absorbance ratio down to 0.3) was tolerable in the singleplex PCR assay with RiACO1 primers. However, in the case of the multiplex PCR assay with these same primers, the DNA purity should be higher than in the case of a singleplex assay. Second, an unusually high variation in the results of DNA concentration measurements combined with high levels of contamination may predict a falsely negative result of a PCR assay. It may also predict altered amplicon melting behaviour. Third, evaluation of real-time PCR-HRM results using melting curves only or melting curves in combination with their similarity values is not sufficient in the case of problematic samples. Fourth, cluster analysis of the normalized amplicon melting curves is a more appropriate method for analysis of real-time PCR-HRM results with problematic samples due to lower ambiguity and better readability/understandability of its output.

## 4. Discussion

Regarding the influence of template DNA contamination on the result of a PCR-based analysis, we have concluded that certain levels of contamination may be tolerable. However, the tolerable levels of contamination will differ with different primer pairs. In the case of VcBHLH003 primers, no DNA isolated from commercial samples was amplified either in singleplex or duplex PCR, while in the case of RiACO1 primers, amplification of DNA from commercial samples was observed (Supplementary Material, Figures S1–S3), and in some cases an accurate result was obtained, even though the template DNA was contaminated.

Similarly to Hugget et al. [40], we were unable to point out a characteristic of the primers, which could be used to predict the susceptibility of the PCR reaction to inhibitors. Regarding the properties and behaviour of template DNA, we observed an association between unusually high variation in concentration measurements and inaccuracy or worsened reproducibility of the results of singleplex real-time PCR-HRM.

Regarding the results of a real-time PCR-HRM analysis with a contaminated template, we show that analysis of such results solely by visual evaluation of normalized melting curves or difference plots may not be sufficient, which is a conclusion similar to one drawn by Nunziata et al. [38]. We also show that the similarity score developed by Sakaridis et al. [41] may be a sufficient measure of similarity between melting curves which are clearly separated into distinct groups when plotted. However, in cases where the result is less clear, a simple calculation of the similarity score is once again not enough and can even lead to inaccurate conclusions, as is demonstrated in this work. We suggest the use of cluster analysis as a solution to this problem, even in cases where the main objective is other than a phylogenetic study. We also demonstrate the use of cluster analysis in a way that does not require sequencing of the amplicons or the use of software with an expensive licence, because the fluorescence values of normalized melting curves are used as input data, as suggested by Farrar and Wittwer [42].

Based on our results, we have several practical suggestions for the design of PCR-based assays for analysis of samples where contamination of template DNA is expected. First, it may be advantageous to test the sensitivity of the primers to inhibition. Second, a primer pair which is tolerant to high levels of contamination in a singleplex PCR setup may be more sensitive to such contamination in a multiplex PCR setup, as was demonstrated here with the RiACO1 primer pair.

The suggestions mentioned above might be helpful in the design of PCR assays intended for analysis of DNAs which are expected to be contaminated, and the DNA isolation protocol (or its parts, such as the use of calcium chloride as an additional way to precipitate polysaccharides) might be helpful in isolating DNA from samples rich in plant material such as blueberry and chokeberry fruit or hibiscus blossoms. This work points out several ways in which problems with PCR-based analysis of plant-based samples may occur and offers possible solutions. While some of these problems are generally well known (such as template DNA contamination), others (such as different sensitivities of various primer pairs to inhibition) are, to our knowledge, not as often mentioned in the literature and therefore might not be thought of when solutions for problems with a PCR-based analysis are sought. This paper offers useful contributions in the form of the above-mentioned suggestions.

## 5. Conclusions

In this paper we focused on the pitfalls of singleplex and multiplex real-time PCR-HRM when it is used to analyze DNA contaminated with potentially inhibitory compounds. We conclude that high levels of contamination combined with an unusually high variation (for the method used) in concentration measurements are predictive of problems with both PCR and HRM analysis and that DNA which shows such behaviour should be discarded and the isolation should be repeated with a different protocol. On the other hand, samples which show high levels of contamination but small variation in concentration measurements may still be successfully amplified and show consistent behaviour between replicates. Second, when the DNA analyzed by real-time PCR-HRM comes from a difficult sample, cluster analysis should automatically follow the visual analysis of melting curves, even if the objective of the experiment is other than a phylogenetic study.

## Figures and Tables

**Table 1:** Composition of commercial teas as declared by the manufacturers.

Tea No.	Composition
1	Rosehips, hibiscus blossoms, seaberry fruit, blackberry leaves 10%, natural aroma, strawberry leaves 5%, mint leaves, liquorice root, raspberry fruit 2%, blueberry fruit 1%
2	Rosehips 25%, seaberry fruit 10%, hibiscus blossoms, chamomile blossoms, blackberry leaves, strawberry leaves, mint leaves, black currant fruit, liquorice root, marigold blossoms
3	Rosehip peels 32%, hibiscus blossoms 26%, apples, orange peel, black currant 7%, blueberries 6%, elder 3%, raspberries 2%
4	Hibiscus blossoms, apple fruit, blackberry leaves, chokeberry fruit 20%, aroma, cinnamon bark, raspberry fruit 2%, camu-camu extract 1%, elderberries, orange pericarp
5	Blueberry fruit 20%, chokeberry fruit 20%, coriander fruit 20%, carrot root 20%, mallow blossom 10%, marigold blossom 5%, elder blossom 5%

**Table 2:** Concentration and purity of DNAs isolated from commercial teas.

Tea No.	c [ng·µL^−1^]	A_260_/A_280_ [-]	A_260_/A_230_ [-]
1	111.6 ± 0.6	1.08 ± 0.00	0.29 ± 0.00
2	124.5 ± 0.5	1.11 ± 0.00	0.30 ± 0.00
3	97.5 ± 13.3	1.25 ± 0.02	0.33 ± 0.00
4	45.7 ± 0.8	1.66 ± 0.01	1.03 ± 0.02
5	88.1 ± 1.2	1.56 ± 0.01	1.23 ± 0.01

**Table 3:** Similarity values for reference samples. B = singleplex PCR mixture with blueberry DNA only, BB = duplex PCR mixture with blueberry and blackberry DNA, and BR = duplex PCR mixture with blueberry and raspberry DNA.

Samples	GCP (%)
B	98.78 ± 0.86
BB	98.06 ± 1.80
BR	99.99 ± 0.01
B × BB	94.35 ± 4.02
B × BR	99.22 ± 0.62
BB × BR	96.87 ± 2.14

**Table 4:** Similarity values for reference samples. D = amplicons from duplex PCR and S = amplicons from singleplex PCR.

	Samples Compared	GCP (%)
1	Blackberry (S)	99.86 ± 0.13
2	Raspberry (S)	99.94 ± 0.04
3	Blackberry (D)	99.70 ± 0.24
4	Blackberry (D) × Blackberry (S)	99.77 ± 0.26
5	Raspberry (D)	99.48 ± 0.51
6	Raspberry (D) × Raspberry (S)	99.73 ± 0.17
7	Blackberry (S) × Raspberry (S)	95.67 ± 1.02
8	Blackberry (S) × Raspberry (D)	96.43 ± 1.91
9	Blackberry (D) × Raspberry (S)	95.63 ± 2.41
10	Blackberry (D) × Raspberry (D)	95.47 ± 2.17

**Table 5:** Similarity values of commercial teas and reference samples. B = blackberry, R = raspberry, T1 = tea 1, T2 = tea 2, T3 = tea 3, and T4 = tea 4.

Sample Pair	GCP (%)	Sample Pair	GCP (%)
B × T1	98.96 ± 0.69	R × T1	99.72 ± 0.18
B × T2	99.18 ± 0.60	R × T2	99.58 ± 0.26
B × T3	90.92 ± 0.44	R × T3	97.44 ± 1.12
B × T4	96.94 ± 0.50	R × T4	99.90 ± 0.08

**Table 6:** Similarity values of commercial teas and reference samples. B = blackberry, R = raspberry, T1 = tea 1, T2 = tea 2, and T4 = tea 4.

Sample Pair	GCP (%)	Sample Pair	GCP (%)
B × T1	99.05 ± 0.64	R × T1	99.24 ± 0.45
B × T2	99.68 ± 0.37	R × T2	97.69 ± 1.24
B × T4	98.38 ± 1.07	R × T4	99.57 ± 0.45

## Data Availability

The original contributions presented in this study are included in the article/Supplementary Material. Further inquiries can be directed at the corresponding author.

## Supplementary Materials

The following supporting information can be downloaded at: https://www.mdpi.com/article/10.3390/mps9020053/s1, Figure S1: Melting peaks of VcBHLH003 amplicons (singleplex PCR); Figure S2: Melting peaks of RiACO1 amplicons (singleplex PCR); Figure S3: Melting peaks of VcBHLH003 and RiACO1 amplicons; Table S1: Primer sequences used in this work; Table S2: Detailed composition of singleplex PCR mixtures; Table S3: Detailed composition of multiplex PCR mixtures for reference samples; Table S4: Detailed composition of multiplex PCR mixtures for commercial samples; File S3: Results of BLAST alignment of the raspberry RiACO1 amplicon to DNA sequences from other plant species.
