# Supplementary material for: Detection of Certain Berries in Difficult Samples by Singleplex and Multiplex Real-Time PCR-HRM: A Case Study of Pitfalls
Source: Methods Protoc. 2026 Apr 1;9(2):53. doi: 10.3390/mps9020053 (PMC13119197; doi:10.3390/mps9020053)
Supplement: Supplementary file 1 [file mps-09-00053-s001.zip › supplementary figures.pdf]

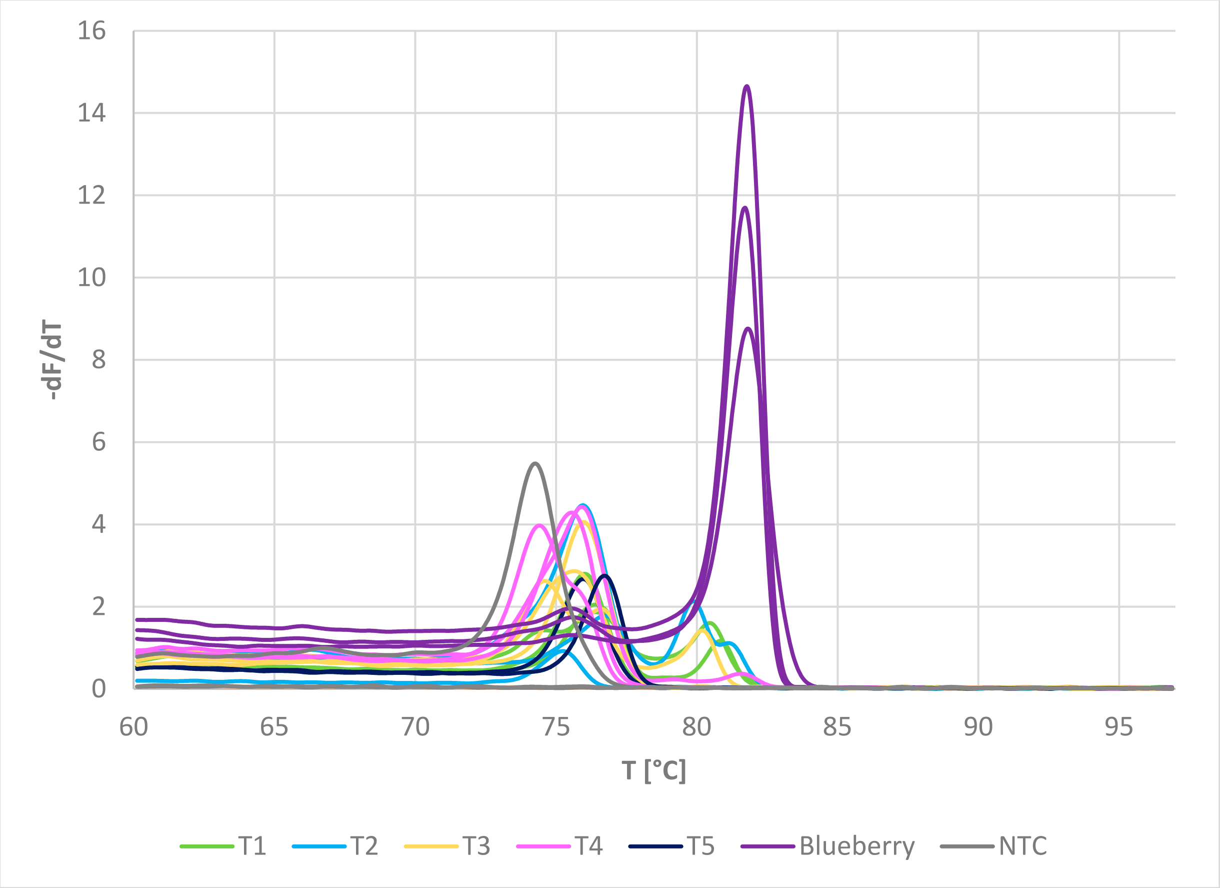

Figure S1: Melting peaks of VcBHLH003 amplicons (singleplex PCR). T1 = tea 1, T2 = tea 2, T3 = tea 3, T4 = tea 4, T5 = tea 5, NTC = no template control

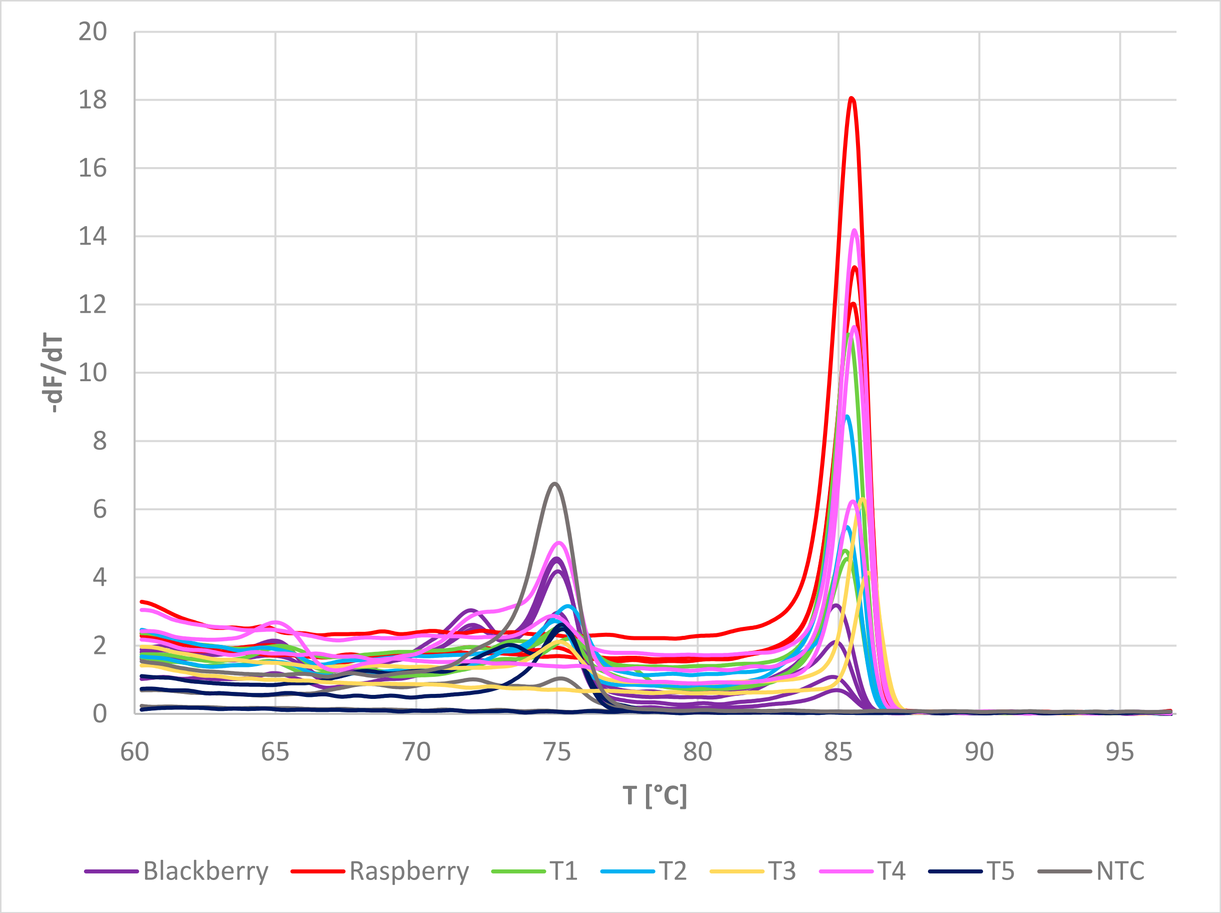

Figure S2: Melting peaks of RiACO1 amplicons (singleplex PCR). T1 = tea 1, T2 = tea 2, T3 = tea 3, T4 = tea 4, T5 = tea 5, NTC = no template control

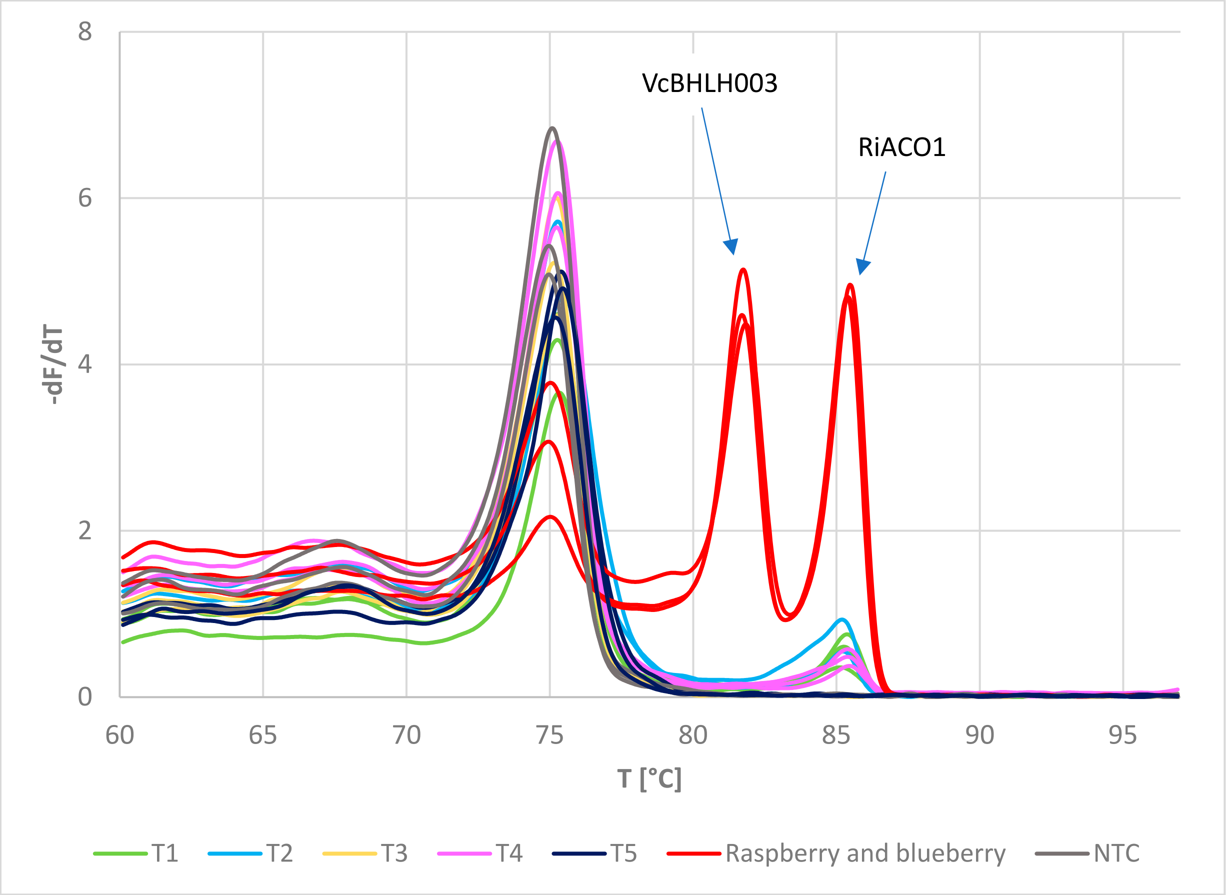

Figure S3: Melting peaks of VcBHLH003 and RiACO1 amplicons. T1 = tea 1, T2 = tea 2, T3 = tea 3, T4 = tea 4, T5 = tea 5, NTC = no template control
