# Supplementary material for: Detection of Certain Berries in Difficult Samples by Singleplex and Multiplex Real-Time PCR-HRM: A Case Study of Pitfalls
Source: Methods Protoc. 2026 Apr 1;9(2):53. doi: 10.3390/mps9020053 (PMC13119197; doi:10.3390/mps9020053)
Supplement: Supplementary file 1 [file mps-09-00053-s001.zip › supplementary tables.pdf]

Table S1: Primer sequences used in this work

Specifity	Name	Sequence	Amplicon length (bp)	Reference
Blueberry	VcVCBHLH003-F	AAATGGATTTGCTGTTATGGGTG	226	(SONG et al. 2017)
	VcVcBHLH003-R	GGAATCATTAGGGAAACTGGGTA		
Raspberry	RiACO1-F	AATTGTTTGGAGCAGAGATTCAAGG	177	(Fuentes et al. 2015)
	RiACO1-R	AAACTCCTTCATCACCTTCCTGTAG		

Table S2: Detailed composition of singleplex PCR mixtures

Primers	VcVCBHLH003	RiACO1
Concentration of each primer in PCR mixture (nM)	200	150
Volume of each primer in PCR mixture (µL)	0.5	0.375
SYTO9 MasterMix (µL)	12.5	12.5
Water (µL)	10.5	10.75
Template DNA (µL)	1.0	1.0

Table S3: Detailed composition of multiplex PCR mixtures for reference samples

Component	Volume (µl)
Water	8.75
SYTO9 MasterMix	12.5
Primers VcVCBHLH003	0.5 each
Primers RiACO1	0.375 each
Blueberry DNA	1.0
Raspberry or blackberry DNA	1.0

Table S4: Detailed composition of multiplex PCR mixtures for commercial samples

Component	Volume (µl)
Water	9.75
SYTO9 MasterMix	12.5
Primers VcVCBHLH003	0.5 each
Primers RiACO1	0.375 each
DNA from commercial tea	1.0
